# Stable Cretaceous sex chromosomes enable molecular sexing in softshell turtles (Testudines: Trionychidae)

**DOI:** 10.1038/srep42150

**Published:** 2017-02-10

**Authors:** Michail Rovatsos, Peter Praschag, Uwe Fritz, Lukáš Kratochvšl

**Affiliations:** 1 Faculty of Science, Charles University, Department of Ecology, Viničná 7, 12844 Praha 2, Czech Republic; 2 Turtle Island, Turtle Conservation Center, Am Katzelbach 98, 8054 Graz, Austria; 3 Museum of Zoology, Senckenberg Dresden, A. B. Meyer Building, 01109 Dresden, Germany

## Abstract

Turtles demonstrate variability in sex determination ranging from environmental sex determination (ESD) to highly differentiated sex chromosomes. However, the evolutionary dynamics of sex determining systems in this group is not well known. Differentiated ZZ/ZW sex chromosomes were identified in two species of the softshell turtles (Trionychidae) from the subfamily Trionychinae and Z-specific genes were identified in a single species. We tested Z-specificity of a subset of these genes by quantitative PCR comparing copy gene numbers in male and female genomes in 10 species covering the phylogenetic diversity of trionychids. We demonstrated that differentiated ZZ/ZW sex chromosomes are conserved across the whole family and that they were already present in the common ancestor of the extant trionychids. As the sister lineage, *Carettochelys insculpta*, possess ESD, we can date the origin of the sex chromosomes in trionychids between 200 Mya (split of Trionychidae and Carettochelyidae) and 120 Mya (basal splitting of the recent trionychids). The results support the evolutionary stability of differentiated sex chromosomes in some lineages of ectothermic vertebrates. Moreover, our approach determining sex-linkage of protein coding genes can be used as a reliable technique of molecular sexing across trionychids useful for effective breeding strategy in conservation projects of endangered species.

Sex determination, the process responsible for the decision whether an individual will develop as a male or a female, is of key importance at both individual and population levels. Surprisingly, particular lineages did not come to a single evolutionary solution of sex determination. Amniotes possess two major sex determination systems^1^: genotypic sex determination (GSD) and environmental sex determination (ESD), usually in the form of temperature-depended sex determination (TSD). In GSD, the sex of an individual is set by its sex-specific genotype, i.e. by the combination of sex chromosomes. On the contrary, in ESD, the sex of an individual is set by environmental conditions, most commonly temperature, during the sensitive period of embryonic development and there are no consistent sex-specific differences in genotypes (e.g. sex chromosomes). The phylogenetic distribution of sex determination systems and recent knowledge on homology of sex chromosomes across amniotes suggests that ESD could be ancestral in this group, while GSD, either as ZZ/ZW or XX/XY system, evolved independently in particular lineages^1^, although this question is far from being solved^2^,^3^.

For a long time, evolutionary stability of sex chromosomes was only documented by molecular methods in endotherms, such as viviparous mammals (ca. 166 My^4^) and birds (ca. 113–138 My^5^,^6^), which led some authors to conclude that the stability of sex chromosomes is related to their effective thermoregulation^7^,^8^. Poikilothermic animals such as reptiles were considered prone to turnovers of sex chromosomes^2^,^9^, particularly due to thermally induced sex reversals^10^,^11^. However, the recent phylogenetic reconstructions of sex determination systems showed that once emerged, GSD, particularly with well-differentiated sex chromosomes, is rather evolutionary stable (‘evolutionary trap hypothesis’)^1^,^12^,^13^ and the larger variability in sex determining systems is probably restricted only to few amniote lineages such as turtles^14^, agamid lizards^15^ and geckos^13^,^16^,^17^, where the variability could be at least partially explained by preservation of the ancestral ESD and several independent turnovers to derived GSD systems^1^. Long-time conservation of sex chromosomes based on molecular evidence has been recently documented in iguanas (Pleurodonta, conserved for ca. 123 My^18^,^19^), caenophidian snakes (ca. 60 My^20^) and lacertid lizards (ca. 70 My^21^,^22^), with the reconstructed ages of the origin of differentiated sex chromosomes and their taxonomic extent approaching the stability of sex chromosomes in viviparous mammals and birds.

Nevertheless, our knowledge on the evolution of sex chromosomes and sex determination systems in reptiles is far from being complete. Identification of sex chromosomes and conclusions about their homology were traditionally conducted based on classical cytogenetic data, which could not discriminate homomorphic or small sex chromosomes (microchromosomes) typical for many reptilian lineages^23^. Moreover, morphology and the stage of heterochromatinization of sex chromosomes is not sufficient for inference of homology of sex chromosome and could lead to misleading conclusions^18^,^19^,^22^,^24^. Recently, molecular-genetic, molecular-cytogenetic and genomic approaches start to flourish in non-avian reptiles, which expanded our knowledge on the gene content and homology of sex chromosomes and facilitated broad phylogenetic comparisons of their sex determination^13^,^25^,^26^,^27^,^28^,^29^.

The aim of the present study is to reveal the evolutionary dynamics of sex chromosomes in softshell turtles (family Trionychidae). Trionychids are known since the Early Cretaceous in Asia^30^,^31^,^32^, representing an ancient lineage of cryptodiran (hidden-necked) turtles. This family includes 32 extant species^33^. Fourteen trionychid taxa are assigned in the IUCN Red list as Vulnerable, Endangered, or Critically Endangered^34^, the black softshell turtle (*Nilssonia nigricans*) is assigned by the IUCN as extinct in the wild with few surviving natural populations^35^,^36^,^37^. On the other hand, some species, especially *Pelodiscus sinensis*, are of increasing commercial value, as several millions of farmed animals are produced per year for food^34^.

A ZZ/ZW sex determination has been identified in two trionychid species: the Chinese softshell turtle, *Pelodiscus sinensis*^38^,^39^, and the spiny softshell turtle, *Apalone spinifera*^26^, both belonging to the subfamily Trionychinae. Members of the second, deeply divergent trionychid subfamily Cyclanorbinae, which diverged approximately 80–120 million years ago^31^,^32^,^40^,^41^ have never been studied with respect to their sex determination mechanism. The sister taxon of trionychids, the Australasian pig-nosed turtle *Carettochelys insculpta*, the sole extant representative of the family Carettochelyidae, possesses TSD^42^.

The sex chromosomes in both studied trionychid species are heteromorphic microchromosomes, with the slightly larger, heterochromatic W chromosome. Both Z and W chromosomes in these species possess the accumulation of the 18S-28S rRNA genes, but the W chromosomes contain much larger number of copies of these genes^26^,^38^. These differences between Z and W chromosomes lead to sexual differences in copy numbers of 18S-28S rRNA genes, which can be quantified by quantitative real-time PCR (qPCR). This approach was validated as a reliable technique for molecular sexing in *A. spinifera*^43^. The partial gene content of the Z chromosome was revealed by physical gene mapping in *P. sinensis*^39^. It was shown that the orthologs of the Z-linked protein-coding genes can be found in the chicken chromosome 15 (GGA15), which points to at least partial homology of the Z of *P. sinensis* and GGA15.

The homology of sex chromosomes in *P. sinensis* and *A. spinifera* was suggested based on the similarity in morphology, heterochromatinization and position of rRNA genes^26^, however, even though the divergence of these two species is rather old (ca. 55–57 My)^41^,^44^, both are phylogenetically closely related as members of the subfamily Trionychinae^45^. Thus, the homology of their sex chromosomes is not informative for sex determination in many softshell turtle lineages. Therefore, using broad phylogenetic sampling of trionychid turtles and including for the first time representatives of the subfamily Cyclanorbinae, we tested the homology of sex chromosomes across trionychid turtles by testing sex-linkage of the genes linked to Z chromosomes in *P. sinensis*. For this purpose, we applied the methodology based on qPCR, which was previously used for testing the homology of sex chromosome in other reptile lineages with both male and female heterogamety^18^,^19^,^20^,^21^,^22^,^46^. Moreover, at the same time, we tested whether this technique based on the comparison of copy numbers of protein-coding genes between males and females is suitable for molecular sexing in the wider phylogenetic spectrum of softshell turtles and we compared it to the technique based on the number of repeats of rRNA genes available for *A. spinifera*^43^.

## Results

**Figure 1:**
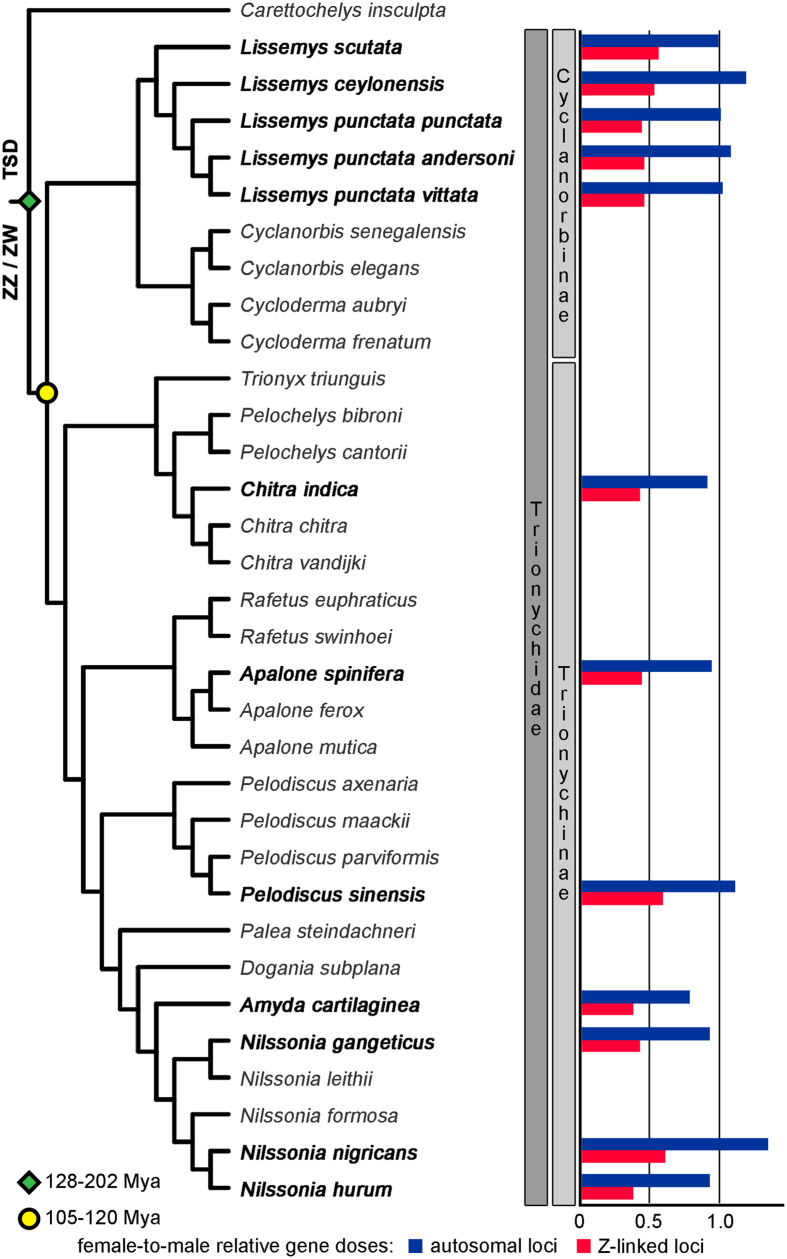
Relative gene dose ratios between female and male genomes in 10 taxa of trionychid turtles. Value 1.0 is expected for autosomal or pseudoautosomal genes, while the value 0.5 is consistent with Z-specificity. The phylogenetic relationships follow ref. 41, the information for the subspecies of *Lissemys punctata* was added from ref. 57. These data suggest that the differentiated ZZ/ZW sex chromosomes were already present in the common ancestor of the extant trionychids and that they have been conserved across the evolution of the group.

In total, six genes were tested for Z-specificity by qPCR in ten trionychid species. As the primers for the qPCR were designed based on *P. sinensis* genomic sequences, some of them did not bind to DNA templates in phylogenetically distant species, or amplified unspecific secondary products, and had to be excluded from the gene dose calculations. However, both autosomal controls and at least three putative Z-specific genes were successfully tested in each species (Fig. 1, Table S3).

The control genes gave consistently gene dosage ratios around 1.0 pointing to their (pseudo) autosomal positions. On the other hand, the successfully tested candidate Z-specific genes had gene dosage ratios around 0.5 in all tested trionychids, which demonstrate Z-specificity of these genes. The stability of Z-specificity of the tested genes in all species from both subfamilies suggests that the ZZ/ZW sex determination system with at least somewhat degenerated W chromosomes was already present in the common ancestor of the family Trionychidae. At the same time, these results show that the qPCR approach based on the comparison of the Z-specific genes is useful for molecular sexing in the wide spectrum of trionychid turtles.

The molecular sexing approach based on the copy-number variation between sexes for the rRNA genes^43^ worked reliably in *A. spinifera*, where the female demonstrate 3.43-times more gene copies than the male. In the original method, *gapdh* was used for normalization of the qPCR values of 18S rRNA. However, we found that *gapdh* primers could amplify unspecific secondary products (e.g. in *Lissemys punctata andersoni*), therefore, we used the single copy gene *rag1* as endogenous control for normalizing of the qPCR values. This method seems to successfully discriminate sexes in nine tested trionychid species, where the females demonstrate 280–1840% of male for 18S rRNA gene copies (Table S3). However, unexpected values were recorded in *Lissemys punctata punctata* (94%) and *Lissemys punctata andersoni* (62%), where females possessed equal or less 18S rRNA gene copies than males (Table S3).

## Discussion

The majority of previously studied turtle species (69 out of 335 recognized turtle species) possess ESD, while GSD has been documented only in 21 species from the families Geoemydidae, Chelidae, Emydidae, Kinosternidae and Trionychidae (for more details see ref. 47). Our study provides data for the presence of the ZZ/ZW sex determining system in further eight turtle species with previously unknown sex determination.

The Z-specificity of the tested genes in all studied species representing both trionychid subfamilies strongly suggests that the common ancestor of the family living ca. 105–120 Mya^32^,^40^,^41^ already possessed ZZ/ZW sex chromosomes with the degenerated W chromosome and that all tested extant trionychid lineages have retained this sex determining system. The sister taxon of trionychids, *Carettochelys insculpta*, possesses ESD^42^. As the families Trionychidae and Carettochelyidae diverged approximately 200 Mya^32^,^40^,^41^,^44^, the emergence of the ZZ/ZW sex chromosomes in trionychids can be tentatively dated between 120 and 200 Mya.

It seems to us that the general impression that endotherms have stable sex chromosomes while ectothermic amniotes unstable sex chromosomes can be at least partially based on our perception based on the old, non-cladistic taxonomy categorizing amniotes to three orders: mammals, birds, and reptiles. Under such classification, two out of three ‘orders’ (birds and mammals) have stable sex chromosomes, although the exceptions are known in viviparous mammals as well^48^,^49^, while a wide array of sex determining systems can be found in ‘reptiles’ and even in their subgroups turtles or ‘lizards’. However, modern birds are just a single, rather young inner lineage of sauropsids with the reconstructed age of sex chromosomes comparable to the age in several ectothermic amniote lineages, e.g. to trionychid turtles as demonstrated here. We hope that among other, our contribution will help to get less biased view on the stability of sex determination mechanisms in vertebrates.

Our study also confirms the suitability of the estimation of the copy number variation of 18S rRNA genes by qPCR for molecular sexing^43^ in *A. spinifera* and several related species from the subfamily Trionychinae, nevertheless, we also noticed individual variability with respect to the copy number of these genes (data not shown). Moreover, the comparison of the copy number of the rRNA genes is not suitable for molecular sexing in all species of trionychids. The accumulations of rRNA genes in the genomes are very dynamic part of the genome even when located on autosomes^24^,^50^,^51^ and probably even more when linked to differentiated sex chromosomes, as repetitive motifs are often evolutionary unstable in degenerated W and Y chromosomes^52^,^53^,^54^. On the other hand, sexual differences in the number of copies of protein coding genes linked to Z and X chromosomes and missing on W and Y chromosomes, respectively, are evolutionary highly stable^18^,^19^,^20^,^21^,^22^,^46^ and their detection is thus suitable for molecular sexing across wide phylogenetic scales using the same primers. Even though our research is primarily oriented towards expanding our understanding on the evolution of sex chromosomes, we believe that our results are valuable for conservation projects as well. One of the big challenges for conservation management is the reliable identification of the sex in animals with poorly developed external sexual characters. Trionychid juveniles are extremely difficult to sex based on external morphology and they often develop visible secondary sexual characteristics only at the age of several years^55^. Our qPCR methodology requiring nondestructive DNA sampling available also in small juveniles thus can help the effective breeding strategy of trionychids.

## Material and Methods

### Material

Tissue and blood samples were collected from ten species of turtles from the family Trionychidae (Table S1). The work was approved by the Ethical Committee of the Czech Republic. The living animals used for blood sampling came from the turtles legally imported to Europe (CITES certificate number DE-KH-050928684, BD 9118332, 26/2013, BD 9118400, 10/2015). All methods were performed in accordance with the relevant guidelines and regulations. DNA was extracted from all samples using the Qiagen DNeasy Blood and Tissue kit (Qiagen, Inc., Valencia, CA) and the concentrations were measured using the ND-1000 spectrophotometer (NanoDrop Technologies, Inc., Rockland, ME). Turtles were phenotypically sexed according to differences in tail length, with males having distinctly longer tails with much more distally located cloacas compared to females.

### Testing the homology of sex chromosomes by qPCR

The Z chromosome of *P. sinensis* contain genes with homologs linked to GGA15. Physical gene mapping^39^ showed that these genes linked to the Z chromosome are missing on its degenerated W counterpart leading to differences in copy gene numbers between sexes. Such sex-specific differences in gene copy number in genomic DNA can be detected by qPCR applied to genomic DNA. Males (ZZ) are expected to possess the double amount of Z-specific gene copies than females (ZW). This qPCR analysis can be performed in other related species to test whether their sex chromosomes are homologous and equally differentiated as those in the model species, i.e. whether the genes that are Z-specific in the model species are also Z-specific in its relatives^18^,^19^,^20^,^21^,^22^,^46^.

Primer pairs for qPCR were designed to amplify 120–200 bp exonic regions of two autosomal (*adarb2, mecom*) and 6 putative Z-linked genes (*anapc7, ccdc92, cux2, ppm1f, sh2b3, tmem132d*), i.e. the genes with homologs linked to GGA15 (Table S2). The genes *rag1* and *mos* were used for normalization of the qPCR values. All primers were designed in Primer-Blast software^56^ based on the sequences from the *P. sinensis* genome^28^. The qPCR analyses with the genomic DNA as the template were carried out using the LightCycler II 480 (Roche Diagnostics). All samples were tested in triplicates. The detailed qPCR protocol and the formula for the calculation of the relative gene dose between sexes have been presented in our previous articles^18^,^19^. The relative female to male gene dosage ratio (r) of 0.5 is expected for Z-specific genes and 1.0 for pseudoautosomal or autosomal genes.

### Test of molecular sexing based on W-linked rRNA repeats

The molecular sexing approach based on qPCR detection of rRNA repeats^43^ was applied for all studied samples. The method was originally developed for *A. spinifera* and it is based on detecting copy-number variation between sexes for the nucleolar organizing region (NOR) by qPCR. The W chromosome in this species has an extensive amplification of 18S rRNA genes compared to the Z chromosome, and as a result, females have approximately four times as many copies of this gene than males^43^. This methodology is analogous to our approach for testing the homology of sex chromosomes by qPCR based on protein-coding genes described above, and thus we could easily apply ‘molecular sexing’ to our turtle samples by tracking the 18S rRNA gene copy variation between samples in parallel (for the primers for 18S rRNA gene see Table S2).

## Additional Information

**How to cite this article**: Rovatsos, M. *et al*. Stable Cretaceous sex chromosomes enable molecular sexing in softshell turtles (Testudines: Trionychidae). *Sci. Rep.* **7**, 42150; doi: 10.1038/srep42150 (2017).

**Publisher's note:** Springer Nature remains neutral with regard to jurisdictional claims in published maps and institutional affiliations.

## Supplementary Material

Supplementary Information
