# Supplementary material for: Stable Cretaceous sex chromosomes enable molecular sexing in softshell turtles (Testudines: Trionychidae)
Source: Sci Rep. 2017 Feb 10;7:42150. doi: 10.1038/srep42150 (PMC5301483; doi:10.1038/srep42150)
Supplement: Supplementary Information [file srep42150-s1.pdf]

Supplementary information to "Stable Cretaceous sex chromosomes enable molecular sexing in softshell turtles (Testudines: Trionychidae)"

MICHAIL ROVATSOS, PETER PRASCHAG, UWE FRITZ and LUKÁŠ KRATOCHVÍL

Table S1: Trionychid specimens used in the study. Samples with museum voucher number were provided from Senckenberg Natural History Collections of Dresden.

Taxon	Sex	Museum voucher number	Origin	Source
Amyda cartilaginea	♂	MTD T 7075	-	pet trade
Amyda cartilaginea	♀	MTD T 7074	-	pet trade
Apalone spinifera	♂	-	-	pet trade
Apalone spinifera	♀	-	-	pet trade
Chitra indica	♂	-	Jamuna River, Bangladesh	captive
Chitra indica	♀	-	Jamuna River, Bangladesh	captive
Lissemys ceylonensis	♂	MTD T 6052	50 km S Colombo, Sri Lanka	wild-caught
Lissemys ceylonensis	♀	MTD T 6053	50 km S Colombo, Sri Lanka	wild-caught
Lissemys punctata andersoni	♂	MTD T 3424	Khulna, Bangladesh	wild-caught
Lissemys punctata andersoni	♀	MTD T 3423	Khulna, Bangladesh	wild-caught
Lissemys punctata punctata	♂	MTD T 6058	Mahabalipuram, Tamila Nadu, India	wild-caught
Lissemys punctata punctata	♀	MTD T 6047	Kotamangalam, Moyyar River, Kerala, India	wild-caught
Lissemys punctata vittata	♂	MTD T 6048	Mangalore, Karnataka, India	wild-caught
Lissemys punctata vittata	♀	MTD T 5369	Goa, India	captive
Lissemys scutata	♂	MTD T 4064	Myanmar	captive
Lissemys scutata	♀	MTD T 4063	Myanmar	captive
Nilssonia gangetica	♂	MTD T 5263	Mahanadi River, Narsinghpur, Odisha, India	wild-caught
Nilssonia gangetica	♀	MTD T 5252	Mahanadi River, Narsinghpur, Odisha, India	wild-caught
Nilssonia hurum	♂	-	-	pet trade
Nilssonia hurum	♀	-	Feni, Bangladesh	captive
Nilssonia nigricans	♂	-	Meghna River, Bangladesh	captive
Nilssonia nigricans	♀	-	Kamakhya, Assam, India	captive
Pelodiscus sinensis	♂	MTD T 14913	Jiangxi, China	wild-caught
Pelodiscus sinensis	♀	MTD T 14912	Jiangxi, China	wild-caught

Table S2. Primers used for the measurement of relative gene dosage by qPCR.

gene name	gene symbol	position in *Pelodiscus sinensis* genome	primer name
recombination activating 1	rag1	NW_005855489.1	rag1_pesi_2
18S major ribosomal RNA subunit	18S rRNA	-	18S_rRNA
adenosine deaminase, RNA specific B2	adarb2	NW_005851920.1	adarb2_pesi_3
MDS1 and EVI1 complex locus	mecom	NW_005857417.1	mecom_pesi_3
cut like homeobox 2	cux2	NW_005857745.1	cux2_pesi_1
SH2B adapter protein 3	sh2b3	NW_005852765.1	sh2b3_pesi_2
transmembrane protein 132D	tmem132d	NW_005854402.1	tmem132d_pesi_1
protein phosphatase, Mg2+/Mn2+ dependent 1F	ppm1f	NW_005858854.1	ppm1f_pesi_1
coiled-coil domain containing 92	ccdc92	NW_005854194.1	ccdc92_pesi_2
anaphase promoting complex subunit 7	anapc7	NW_005858868.1	anapc7_18

forward primer	reverse primer	amplicon size
CAAAATGCTATGAAATGGAGGATGT	TCTTCTAAACTCTGCCCTGGA	136
GAGTATGGTTGCAAAGCTGAAA	CGAGAAAGAGCTATCAATCTGT	144
AAACGGCGAGAGGATTGGGA	TTCATAGGGAGAGTTGAGGCGTG	138
AACAAACAAAAGTCAAAGTCCCC	ACATCATCAAGGTCACTACTCTCT	195
AGGTCTCTCTGAACTCGCCC	GTGCCTTCGCTGGATGTTTGA	200
GACTATCGGGAGACCAGCA	CAGATGGGTGAGAGAGAAGTGT	182
CGCTGAACCCTCCGACTCA	CCCAATCAAAACCTGTCCACCT	145
AGATGGTTTCAAGGCAGCAGAG	CTGAGGTTTTTAGGGTCTCCCAG	141
ACGCACTTCAGGAGAATCACAAA	ATGCTTTCGGACCACTTTACTTC	163
TAGGTTTGTTGTCGCTCTCAGT	GGCTCTTGTGTTATCTCCTGTGT	143

Table S3. Relative gene dose ratios (r) between female and male genomes.
The symbol "x" means that the primer was tested, but the amplification was not successful (e.g. due to secondary product).

primer pair	assumed position in trionychid genomes	*Lissemys scutata*	*Lissemys ceylonensis*	*Lissemys punctata punctata*
**18S_rRNA**	Z and W chromosomes	2.80	5.15	0.94
**adarb2_pesi_3**	(pseudo)autosomal	1.03	1.20	1.02
**mecom_pesi_3**	(pseudo)autosomal	0.98	1.18	
**Average (pseudo)autosomal values:**		1.01	1.19	1.02
**cux2_pesi_1**	Z-specific	0.58	0.51	0.39
**sh2b3_pesi_2**	Z-specific	0.50	0.54	0.36
**tmem132d_pesi_1**	Z-specific	x	x	x
**ppm1f_pesi_1**	Z-specific	0.55	0.48	0.60
**ccdc92_pesi_2**	Z-specific	0.60	0.63	0.43
**anapc7_18**	Z-specific	x	x	x
**Average Z-specific values:**		0.56	0.54	0.45

Lissemys punctata andersoni	*Lissemys punctata vittata*	*Chitra indica*	*Apalone spinifera*	*Pelodiscus sinensis*	*Amyda cartilaginea*
0.69	4.55	3.68	3.43	4.40	11.00
1.10	1.04	1.10	1.02	1.16	0.86
1.07		0.76	0.90	1.08	0.72
1.09	1.04	0.93	0.96	1.12	0.79
0.43	0.41	0.54	x	0.92	0.40
0.52	0.50	0.51	x	0.66	0.38
x	x	0.43	0.51	0.45	0.39
0.48	0.47	0.37	x	0.56	0.35
0.46	0.49	0.36	0.43	0.54	0.40
x	x	0.39	0.42	0.51	x
0.47	0.47	0.43	0.45	0.61	0.38

Nilssonia gangetica	*Nilssonia nigricans*	*Nilssonia hurum*
3.60	18.40	3.20
1.08	1.40	0.92
0.78	1.29	0.96
0.93	1.35	0.94
0.39	0.63	x
0.37	0.54	0.42
0.46	0.51	0.38
0.49	0.62	0.41
0.48	0.74	0.38
0.47	0.61	0.35
0.44	0.61	0.39
